# Emerging gene editing strategies for Duchenne muscular dystrophy targeting stem cells

**DOI:** 10.3389/fphys.2014.00148

**Published:** 2014-04-21

**Authors:** Carmen Bertoni

**Affiliations:** Department of Neurology, David Geffen School of Medicine, University of California Los AngelesCA, USA

**Keywords:** muscle stem cell, satellite cells, DMD, *mdx*, gene repair, gene correction, ssODN, dystrophin

## Abstract

The progressive loss of muscle mass characteristic of many muscular dystrophies impairs the efficacy of most of the gene and molecular therapies currently being pursued for the treatment of those disorders. It is becoming increasingly evident that a therapeutic application, to be effective, needs to target not only mature myofibers, but also muscle progenitors cells or muscle stem cells able to form new muscle tissue and to restore myofibers lost as the result of the diseases or during normal homeostasis so as to guarantee effective and lost lasting effects. Correction of the genetic defect using oligodeoxynucleotides (ODNs) or engineered nucleases holds great potential for the treatment of many of the musculoskeletal disorders. The encouraging results obtained by studying *in vitro* systems and model organisms have set the groundwork for what is likely to become an emerging field in the area of molecular and regenerative medicine. Furthermore, the ability to isolate and expand from patients various types of muscle progenitor cells capable of committing to the myogenic lineage provides the opportunity to establish cell lines that can be used for transplantation following *ex vivo* manipulation and expansion. The purpose of this article is to provide a perspective on approaches aimed at correcting the genetic defect using gene editing strategies and currently under development for the treatment of Duchenne muscular dystrophy (DMD), the most sever of the neuromuscular disorders. Emphasis will be placed on describing the potential of using the patient own stem cell as source of transplantation and the challenges that gene editing technologies face in the field of regenerative biology.

## Introduction

The discovery of dystrophin as the gene responsible for Duchenne muscular Dystrophy (DMD) has enabled researchers to identify several of the genes linked directly or indirectly to dystrophin and to correlate defects in those genes to many of the different forms of muscular dystrophies (Monaco et al., 1986; Hoffman et al., 1987; Koenig et al., 1987). Despite the diversity in phenotypic and pathological manifestation of various forms of muscular dystrophies identified to date, many display common symptoms. Characteristic is the progressive loss of muscle mass which has been attributed, at least in part, to the inability of muscle stem cells to efficiently regenerate tissue lost as the result of the disease. Great progress has been made toward the identification of therapies for DMD. Potential approaches range from gene augmentation strategies using viral or plasmid vectors aimed at restoring dystrophin expression to upregulation of genes that could be used to overcome the lack of expression of the defected gene. While some of these approaches have sown efficacy, the results obtained to date have also expounded limitations in the clinical applicability of therapeutic applications to DMD. In particular, the progressive loss of expression of the therapeutic gene observed following treatment have clearly indicated that targeting mature myofibers alone is not sufficient to preserve the beneficial effects achieved by the therapeutic approach (Bertoni et al., 2006; Kayali et al., 2010). Critical to the development of effective strategies to treat muscle disorders is the optimization of approaches targeting muscle stem cells and capable of regenerating tissue lost as the result of the disease or as the result of normal muscle turnover.

Muscle stem cells are classically defined as undifferentiated cells characterized by their unique ability to activate in response to specific stimuli and self-renew. Daughter cells originated by the division of stem cells can either retain their stem cell identity or differentiate into a more committed lineage capable of producing new muscle tissue or of fusing with preexisting myofibers to repair damage ones. Among the different types and subtypes of muscle stem cells identified to date, satellite cells (SCs) are probably the most studied. Since their initial identification (Mauro, 1961) studies have clearly demonstrated that SCs are composed by an heterogeneous population of muscle stem cells distinguishable based on their gene expression signatures, their ability to commit into a specific myogenic lineage, their capacity to assume non-myogenic cell-fate and differences in their ability to activate in response to specific queues (Schultz, 1996; Seale et al., 2000; Ono et al., 2010; Bentzinger et al., 2012; Brack and Rando, 2012; Yin et al., 2013). Other types of stem cells capable of assuming a myogenic cell fate and of regenerating muscles have been described. Those include bone marrow stem cells (Ferrari et al., 1998; Bittner et al., 1999; Gussoni et al., 1999) muscle side population (SP) cells (Gussoni et al., 1999; Asakura et al., 2002; Rivier et al., 2004; Doyle et al., 2011), muscle-derived stem cells (Lee et al., 2000), mesangioblasts (Minasi et al., 2002; Sampaolesi et al., 2003; Galli et al., 2005; Morosetti et al., 2006), pericytes (Dellavalle et al., 2007; Peault et al., 2007), embryonic stem cells (ESCs) (Bhagavati and Xu, 2004; Barberi et al., 2007; Darabi et al., 2009; Filareto et al., 2012) and induced pluripotent stem cells (iPSCs) (Chang et al., 2009; Kazuki et al., 2010; Darabi et al., 2012; Tedesco et al., 2012).

Therapeutic approaches to muscle disorders and targeting stem cells have focused primarily on demonstrating the feasibility of restoring dystrophin expression following transplantation of cells isolated from healthy donors. Proof-of-concept studies have been performed in immunosuppressed *mdx* mice that have been used as models for DMD (Coulton et al., 1988; Sicinski et al., 1989). Some success has been achieved using transplantation of freshly isolated SCs (Collins et al., 2005; Boldrin et al., 2009, 2012) or subpopulations of SCs isolated using fluorescence activate cell sorting (FACS) which have been used primarily to demonstrate the existence of distinguished populations of SCs with regenerative capacity and capable of self-renewing (Cerletti et al., 2008; Sacco et al., 2008; Rocheteau et al., 2012).

Despite the encouraging results obtained to date in the field, issues still remain that may hamper the applicability of cell-mediated regenerative approaches to muscle disorders. Among those, is the need to use heterologous sources for the transplantation procedure and the risk of immune rejection associated with their use. The issue of immune response could be overcome by the use of reprogrammable stem cells capable of differentiate into muscle progenitor cells such as human ESCs human iPSCs (Darabi et al., 2012) or mesoangioblasts (Tedesco et al., 2012) isolated directly from the patient and that have been genetically modified to express dystrophin or other therapeutically relevant genes. Among the technologies currently being investigated for the treatment of DMD, gene editing is perhaps the most exciting as it offers the possibility to correct a genetic defect at the source of the problem, the DNA and can therefore promise to restore a completely functional protein. Critical to the success of gene editing strategies for muscle disorders is to target cells capable of retaining the stem cells properties to ensure that the beneficial effects achieved by the gene correction process is maintained over time. As such, the use of muscle stem cells capable of self-renewing is likely to have advantages over other type of cells, namely due to their ability to actively participate to the regenerative process over prolonged periods of time with virtually little or no loss of regenerative potential.

**Figure 1:**
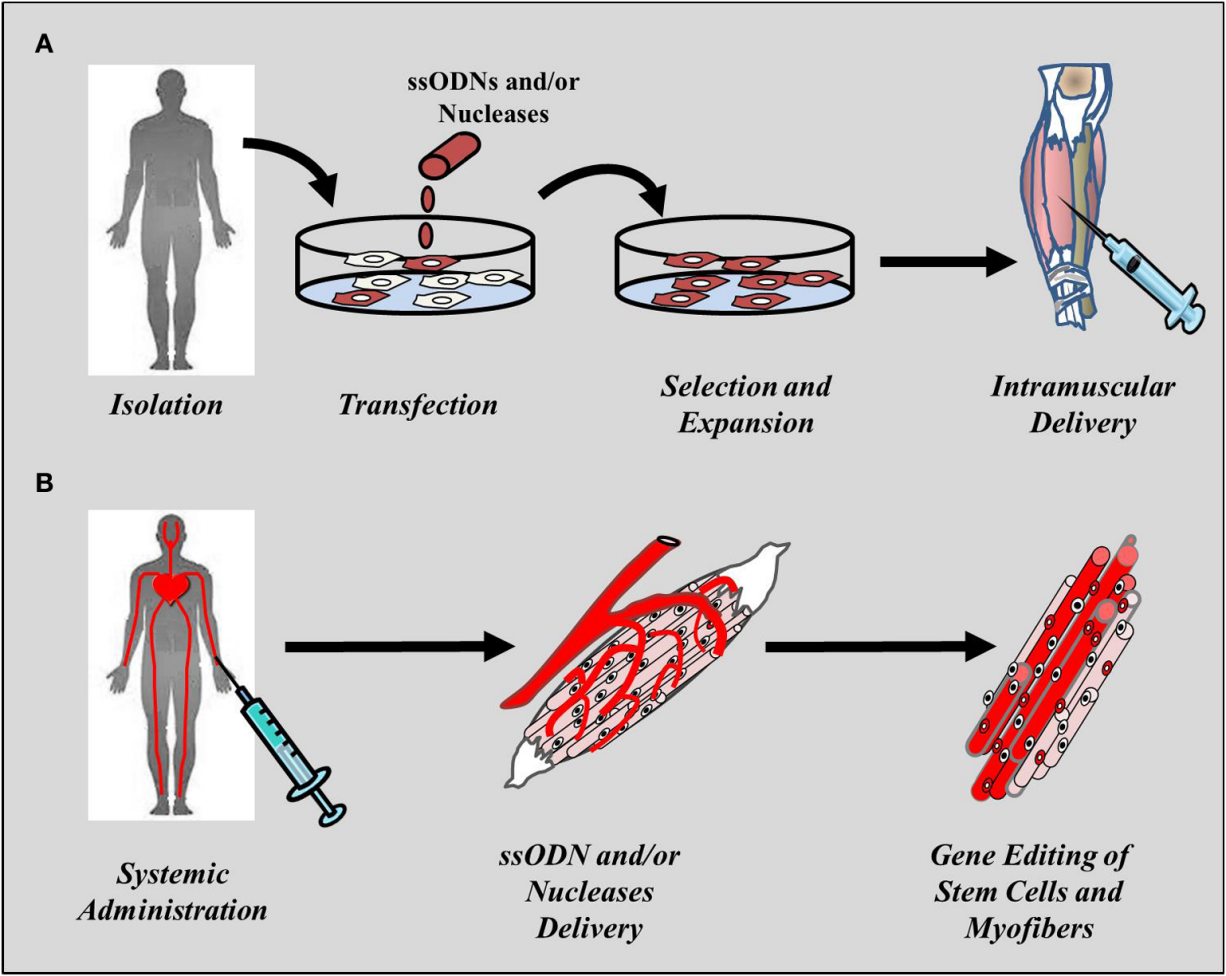
**Cell-mediated regenerative approaches to muscle disorders**. Gene editing strategies in muscle stem cells are aimed at either correcting the genetic disorder *ex vivo* or at delivery the therapeutic agent *in situ* following systemic administration. **(A)** *Ex vivo* approaches requires harvesting of cells from the patient or healthy donor, reprogramming in cases were the cells being employed are not muscle-derived, editing using the targeting tools, selection of the cells undergone repair and expansion prior to delivery into muscle through local, or systemic administration. **(B)** Delivery of the gene editing tools into muscle typically uses the circulatory system and can employ intraperitoneal, subcutaneous, intra-arterial, or intravenous administration depending on the physico-chemical, and pharmacological properties of the therapeutic agent being employed. The method is much less invasive than direct intramuscular injection and has the potential of targeting a large number of muscles simultaneously.

The use of gene editing in muscle stem cells for therapeutic purposes can be divided into two major applications: strategies aimed at targeting muscle stem cells *ex vivo* that can be used for transplantation purposes and strategies aimed at targeting and correcting stem cells *in situ* following systemic administration of the therapeutic agent into the patient's own stem cells, mainly SCs (Figure 1). Both approaches present pros and cons as discussed in more detail below (see Drawbacks and limitations of gene editing mediated by ODNs and endonucleases). Among the hurdles that will need to be overcome before cell-mediated therapies can be brought into the clinic is the need to target a large number of muscles for the therapy to be clinically relevant. Nonetheless, the use of gene editing strategies in muscle stem cells is likely to become a valid therapeutic alternative to gene augmentation therapies. This review will provide an overview of the progress made in the past decade toward the development of gene editing tools for the treatment of DMD and the current state-of-the art of technologies aimed at permanently correct the genetic defect in muscle progenitor cells and stem cells.

## Oligonucleotide-mediated gene correction

**Figure 2:**
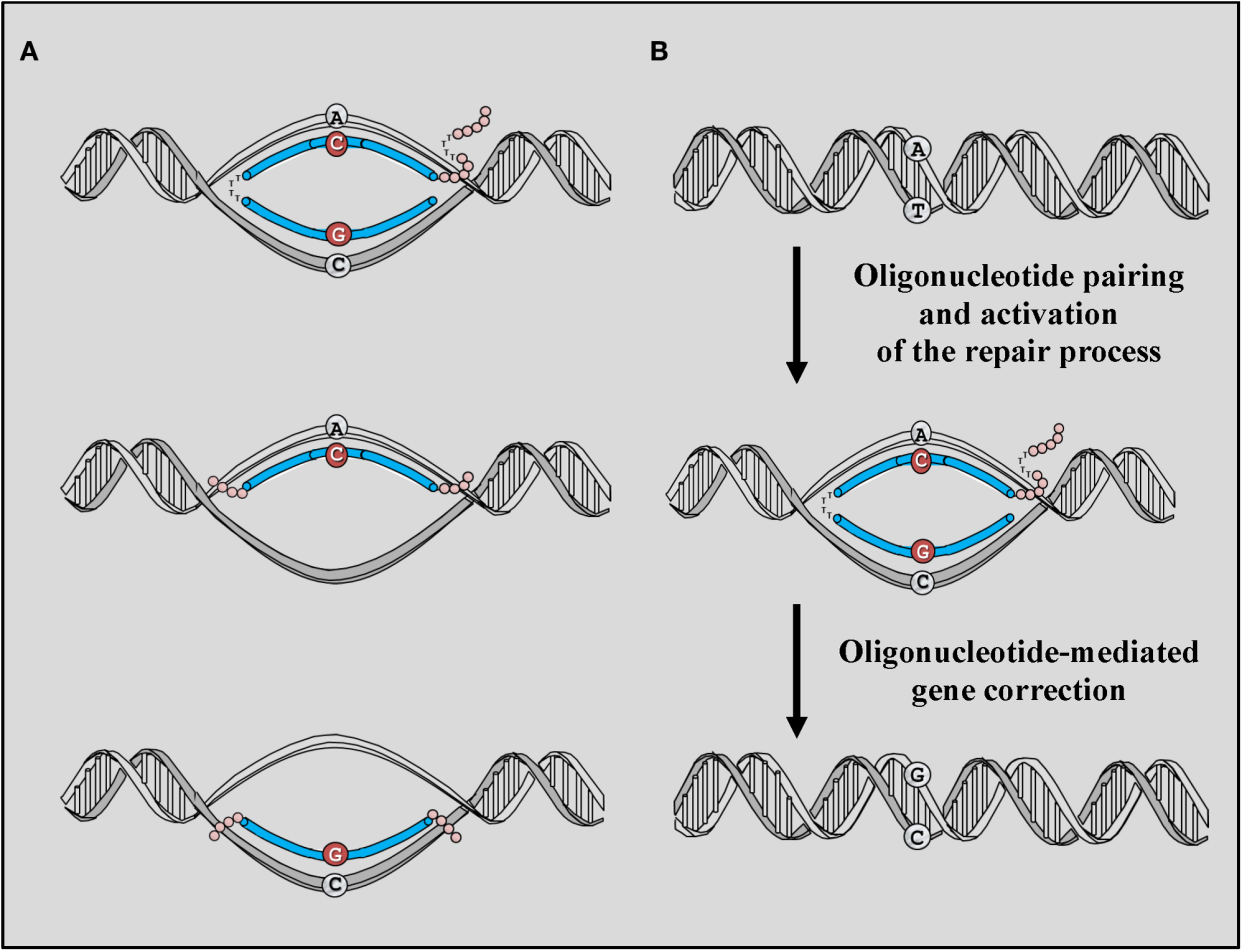
**ODN-mediated gene editing. (A)** The basic structure of targeting ODNs consists of a stretch of DNA perfectly homologous to the region of the gene that is targeted for correction with the exception of a single base mismatch. A chODN contains a complimentary region composed of 2′-O-methyl RNA interrupted by a pentameric block of DNA bases while ssODNs consist of unmodified DNA bases and can be complimentary to the coding strand or complimentary to the non-coding strand of the gene targeted for correction. Phosphorothioate (PS) bases are added to the each end of the ODNs to increase their stability to endonucleases. **(B)** The correction process is activated by the pairing of ODN with the genomic sequence targeted for repair. Recognition of the mismatch present on the ODNs activates innate repair mechanisms naturally present in the cell nuclei and capable of directing the correction based on the information provided by the ODN template. The process require the presence of protein such as RecA and MSH2 capable of recognizing and correct the mismatch.

Different areas of investigations have focused on the possibility of using oligodeoxynucleotides (ODNs) as therapeutic vectors. First among those, the success obtained using homologous recombination (HR) technologies, an approach that has been employed extensively to generate animal models to study disease mechanisms (Capecchi, 1989). However, the low frequency of HR and the high frequency of non-homologous integration of such constructs have clearly evidenced serious limitations in the applicability of this approach for the treatment of inherited diseases and have prompted the development of new, safer vectors capable of activating repair mechanisms other than HR and capable of introducing single base pair (bp) alterations at the genomic DNA without the need of integrating into the genome. Gene editing mediated by ODNs generally employs short (less than 100 nucleotides) synthetic DNA or RNA sequences homologous to the region of the genomic DNA targeted for repair. The technology differ substantially from that employing antisense oligonucleotides (AONs), that also uses oligonucleotides, but that act at the messenger RNA (mRNA) level to block and therefore redirect splicing of the mRNA to produce shorter although still functional proteins (Arechavala-Gomeza et al., 2012). Gene editing mediated by ODNs takes advantage of innate repair mechanisms present in the cell and responsible for maintenance of chromosome integrity. The process requires multiple steps which begin with the pairing of the ODN with the region of the genomic DNA targeted for repair, recognition of the mismatch on the targeted base, excision of the mutation, and insertion of the desired base (Figure 2).

### Evolution of ODNs for gene editing purposes

The initial vectors employed for gene editing strategies consisted of chimeric DNA/RNA ODNs (chODNs) made of 68 residues which were originally given the name of chimeraplasts. The vector contained both RNA and DNA residues complementary to the region of the genomic DNA targeted for repair and flanked by 2′-O-methylated RNA residues which were used to increase resistance to RNase H activity (Figure 2A). To increase stability and maintain their secondary structure, ODNs were designed to contain at their 3′ and 5′ ends polythymidine hairpins and a 3′ tag containing a clamp made of guanidine and cytosine residues (Figure 2A) (Cole-Strauss et al., 1996; Kren et al., 1999a,b; Bertoni, 2008). These chODNs were designed to pair with both strands of the gene targeted for repair and to activate DNA mismatch repair (MMR) mechanisms though the recognition of the single base mismatch present on the ODN (Figure 2B). The activation results in the conversion of the targeted base at the genomic level using the information provided by the chODN. Since their first application, chODNs have been investigated in their ability to target and induce genomic modifications in a number of different cell types and have been successfully applied in both eukaryotic and prokaryotic cells (Cole-Strauss et al., 1996, 1999; Kren et al., 1998, 1999b; Beetham et al., 1999; Zhu et al., 1999, 2000; Bartlett et al., 2000; Rando et al., 2000; Rice et al., 2000, 2001; Tagalakis et al., 2001; Igoucheva and Yoon, 2002).

The initial studies exploring the feasibility of using chODNs for the treatment of muscular dystrophies were performed in the *mdx* mouse model for DMD (Rando et al., 2000; Bertoni and Rando, 2002; Bertoni et al., 2003). A chODN designed to target and correct the single point mutation present in exon 23 of the dystrophin gene was shown to restore dystrophin expression in both muscle precursor cells in culture (Bertoni and Rando, 2002) as well as *in vivo* following direct intramuscular injection (Rando et al., 2000). Importantly, correction was demonstrated to be stably inherited in dividing cells and to result in restoration of full-length dystrophin expression. When administered intramuscularly at high doses, the chODN was able to distribute into approximately 40% of the SCs present in the muscle (Bertoni and Rando, 2002). Once explanted, SCs that had taken up the chODN targeting the *mdx* mutation, were shown to proliferate and expand *in vitro* to produce myoblasts capable of differentiating and to form myotubes which expressed full-length dystrophin. The level of gene repair detected in those cells remained substantially lower than that achieved in muscle progenitor cells transfected with the targeting chODN in culture demonstrating the presence of intrinsic differences in the ability of SCs to undergo gene repair compared to myoblasts (Bertoni and Rando, 2002). Nonetheless, the results clearly indicated the feasibility of using ODNs to target and correct SCs and demonstrated for the first time the possibility of targeting SCs *in situ* following delivery of ODNs. Studies in the GRMD have confirmed the feasibility of using chODNs to correct defects in the dystrophin gene in larger animals (Bartlett et al., 2000).

A key advancement in vector development was the discovery that ODNs made of single stranded DNA sequences (ssODNs) were as efficient as chODNs in directing the gene correction process (Gamper et al., 2000a,b) rendering this technology widely available to virtually any laboratory interested in exploring its potential application in different prokaryotic and eukaryotic systems and for different applications (Igoucheva et al., 2001, 2008; Dekker et al., 2003, 2006; Nickerson and Colledge, 2003; Pierce et al., 2003; Radecke et al., 2004; Bertoni et al., 2005; Olsen et al., 2005b; Sorensen et al., 2005; Aarts et al., 2006; Morozov and Wawrousek, 2008; Disterer et al., 2009). ssODN can either be complementary to the leading strand of the genomic loci or complimentary to the lagging strand (Figure 2A). In muscle, gene correction mediated by ssODN has been assessed using the *mdx^5cv^* mouse. In this model, an A-to-T transversion in exon 10 of the dystrophin gene creates a cryptic splice site recognized by the splicing machinery. Thus, the mRNA of the dystrophin gene is aberrantly spliced causing total absence of dystrophin (Im et al., 1996). The use of the *mdx^5cv^* mouse allows to precisely quantitate frequencies of gene repair achieved at both, the genomic DNA and mRNA levels, making this animal model particularly valuable (Bertoni et al., 2005; Kayali et al., 2010). Results clearly indicated that ssODNs complementary to the coding strand were as effective as chODN in correcting the dystrophin gene defect in *mdx^5cv^ in vitro* as well as *in vivo*. A strand bias was observed in the correction abilities of linear DNA ODNs depending on whether the ssODNs were targeting the coding or the non-coding strand of the dystrophin gene, suggesting that transcription may influence the ability of ssODNs to induce the genetic alteration at the chromosome level (Bertoni et al., 2005). Differences in strand bias observed by others seem to confirm the implication of transcription in the processes that take place in gene repair mediated by ssODNs (Igoucheva et al., 2001, 2003; Liu et al., 2001, 2002).

In recent years, studies have focused primarily on identifying new generation of ssODNs that could promote more efficiently the repair process in an effort to increase the frequencies of gene repair to levels that would be considered therapeutically relevant. Some success has been obtained by increasing the stability of the ODNs. The use of ssODNs containing 2′-*O*-methyl RNA residues (Igoucheva et al., 2001; Nickerson and Colledge, 2003), PS linkages (Liu et al., 2002; Olsen et al., 2005b), or Locked Nucleic Acid (LNA) bases (Parekh-Olmedo et al., 2002) at their extremities have been shown to increase targeting frequencies. Several studies have also demonstrated that gene repair can be enhanced by synchronizing the cells in the S phase of the cell cycle or by reducing the rate of replication fork progression (Brachman and Kmiec, 2004; Ferrara and Kmiec, 2004; Ferrara et al., 2004; Olsen et al., 2005a). Promising results have been achieved in muscle using ssODNs made of peptide nucleic acids (PNAs) bases (Kayali et al., 2010). PNAs are DNA mimics capable of forming stable duplex structures with Watson-Crick complementary DNA or RNA with a higher binding affinity than that of DNA/DNA or DNA/RNA duplexes made of unmodified bases (Nielsen, 2005). In general, ssODNs stretching from 12 to 18 nucleotides are sufficient to allow strong duplex formation with their complementary DNA or RNA sequences and to distinguish single base mutations. Kayali et al demonstrated that ssODNs made of PNA (PNA-ssODNs) containing the appropriate mismatch were capable of targeting and correcting the *mdx^5cv^* mutation in the dystrophin gene more efficiently than ssODNs made of unmodified bases (Kayali et al., 2010). Expression of full-length dystrophin was sustained for up to four months after injection although correction was shown to decrease over time as the result of normal muscle turnover (Kayali et al., 2010). These results were particularly important to the field fo gene editing for DMD because provided the first evidence of how correction of the genetic defect in mature myofibers alone is not sufficient to guarantee long lasting effects and paved the way for subsequent studies aimed at studying the feasibility of targeting and correcting SCs and their therapeutic relevance for the treatment of DMD.

### Mechanisms of action of ODN-mediated gene repair

It is believed that chODNs and ssODNs act through similar mechanisms and that the repair process involves multiple steps (Gamper et al., 2000a,b; Igoucheva et al., 2004). The first steps requires the annealing of the ODN to the region of the genomic DNA targeted for repair (Liu et al., 2003; Jensen et al., 2011; Papaioannou et al., 2012). Pairing leads to the formation of a heteroduplex between the ssODN and the double-stranded target site (Figure 2B) (Bertoni, 2005, 2008; Engstrom et al., 2009). Msh2, a member of the family of proteins involved in the MMR mechanism has been shown to inhibit the repair process, potentially by preventing recombination between the ODN and the targeted genomic sequence, a phenomenon known as heteroduplex rejection (Dekker et al., 2003; Pierce et al., 2003; Aarts et al., 2006; Maguire and Kmiec, 2007; Igoucheva et al., 2008; Papaioannou et al., 2009). Furthermore, a two- to three-fold increase in frequencies of gene repair has been reported recently in primary cultures isolated from *mdx^5cv^* muscle transfected with targeting ssODNs in conjunction with a siRNA designed to transiently downregulate Msh2 expression supporting the implication of the MMR as one of the mechanisms that controls ssODN-mediated gene repair in muscle cells (Maguire et al., 2009). Interestingly, the authors also failed to detect an effect when Msh2 was downregulated in purified myoblasts maintained in culture for prolonged period of time suggesting that the MMR may not be the only mechanism involved in the correction process in myoblasts and that culturing conditions of these cells may influence the repair process.

The second phase implicated in the correction process mediated by ODNs, involves the activation of the repair process. Some studies have implicated the HR pathway through homology-directed repair (HDR) and non-homologous end-joining (NHEJ) mechanisms as one of the mechanisms responsible for the correction induced at the genomic level through evidences that demonstrate that a portion of the ODN becomes integral part of the genomic DNA (Radecke et al., 2006; Aarts and te Riele, 2010). However, it is evident that mechanisms other than HR may be involved in the process catalyzed by ODNs. Among those, the nucleotide excision repair (NER) pathway appear to play a role and it was demonstrated that two of the proteins involved in this repair pathway, XPG and ERCC4, are required to facilitate ssODN-mediated gene repair, whereas components in the NHEJ pathway was found to inhibit the correction process (Igoucheva et al., 2006).

Recent studies aimed at improving the specificity and efficacy of ssODNs in directing single base alterations at the genomic level have also evidenced the possibility of recruiting repair mechanisms other than HR and NER (Bertoni et al., 2009). The approach involves the use of ssODNs containing methyl-CpG sequences and capable of activating the base excision repair (BER) mechanism through the recruitment of the methyl-CpG binding domain protein 4 (MBD4) also known as MED1. MBD4 is thought to be responsible for maintaining genome integrity by recognizing G:T or G:U mismatches at m^5^CpG sites on double-stranded DNA (Bellacosa et al., 1999; Hendrich et al., 1999). *In vitro* studies have demonstrated that MBD4 can efficiently recognize and hydrolyze G:T or G:U mismatches at hemi-methylated m^5^CpG sites as well as G:T and G:U mismatches in non-methylated CpG sequences (Hendrich et al., 1999). The introduction of the DNA mismatch is recognized by DNA glycosylases which excise the damaged base from the genomic DNA creating an apurinic/apyrimidinic site (AP site). The DNA strand is subsequently processed by specific endonucleases and ligases to direct the addition of a new cytosine at the AP site and to complete the repair process using the ssODNs as template (Bertoni et al., 2009). This new generation of ssODNs was shown to efficiently correct a single point mutation introduced in a GFP reporter system which was stably transfected in myoblasts. The drawback of using methyl-CpG-modified ssODNs is represented by the sequence specificity of the mutations that can be targeted by this approach which limits its broad applications in all genetic defects (Bertoni et al., 2003, 2009).

### Therapies for DMD using ODNs targeting stem cells

The feasibility of using ssODNs to correct gene defects in SCs and restore full-length dystrophin expression has recently been demonstrated by Nick-Ahd et al. In the study, the authors isolated SCs from the *mdx^5cv^* mouse and transfected PNA-ssODNs targeting the *mdx^5cv^* mutation prior to engraftment into immunocompromised *mdx*/nude mice (Nik-Ahd and Bertoni, 2014). Clusters of dystrophin-positive fibers were clearly detected immediately following transplantation and expression of dystrophin, resulting from the contribution of donor-derived SCs that had undergone gene repair, were shown to increase over time. The work represent the first evidence of the feasibility of inducing *ex vivo* gene repair of SCs without compromising the ability of isolated cells to self-renew following transplantation (Nik-Ahd and Bertoni, 2014).

More recently, gene editing strategies mediated by ssODNs has been extended to iPSCs isolated from human skin fibroblasts of two patients affected by type I spinal muscular atrophy (SMA). SMA is an autosomal recessive genetic disorder caused by a genetic defect in the survival motor neuron 1 (*SMN1*) gene, which encodes SMN. Loss of SMN protein is thought to be responsible for the progressive loss of motor neurons which is paralleled by the progressive muscle wasting characteristic of SMA patients (Brzustowicz et al., 1990; Lefebvre et al., 1995; Coovert et al., 1997). Corti et al used a 75 bp ssODN was used to target and redirect splicing of the *SMN2* gene, a gene paralogous to *SMN1* and to induce expression of a protein similar to SMN1. This strategy has previously been shown to partially rescue motor neuron loss in animal models and is considered a valuable approach to treat the disease in patients (Lefebvre et al., 1997; Le et al., 2005). Correction mediated by ssODNs targeting the *SMN2* gene was achieved in approximately 4% of the transfected cells suggesting frequencies of gene repair similar to those observed in muscle culture and demonstrating that iPSCs are equally amenable to gene repair than myoblasts and SCs (Bertoni et al., 2005, 2009; Maguire et al., 2009; Kayali et al., 2010; Nik-Ahd and Bertoni, 2014). Importantly, when transplanted into the spinal cords of 1-day-old SMA mice, donor-derived motor neuron engrafted in the anterior spinal cords of transplanted mice and ameliorated defects in neuromuscular function in SMA mice (Corti et al., 2012). These results are particularly encouraging as they represent the first evidence of how gene editing mediated by ssODNs could have a clinical applicability to disorders other than DMD demonstrating that the field of gene repair is slowly but steadily growing toward the development of clinical applications for the treatment of different neuromuscular disorders.

## Nuclease-mediated gene editing

During the past 10 years, the field of gene editing has witnessed a tremendous growth in the number of laboratories interested in developing corrections strategies using engineered nucleases. These nucleases are artificial restriction enzymes that can be designed to target virtually any site in the genome. Their use has enabled routine reprogramming of prokaryotic and eukaryotic systems for a variety of applications ranging from site-directed mutagenesis of bacterial systems, generation of animal models to study diseases, or simply proof-of-concept studies to demonstrate the specificity of a biological system. The ability of nucleases to recognize specific sequences in the genome and introduce a cleavage at specific sites has been known for almost two decades, but this technology has boomed only recently. This rapid growth is in part due to the crescent interest of commercial sources in developing new products that could be brought to the market and in part to the recognition of public and government sources of the potential that this application could have in basic and translational biology. More recently, nucleases have moved into preclinical and clinical applications for numerous diseases and three phase I and phase II clinical trials are on their way in Human Immunodeficiency Virus (HIV) patients (Tang, 2013a,b; Tebas and Stein, 2013).

**Figure 3:**
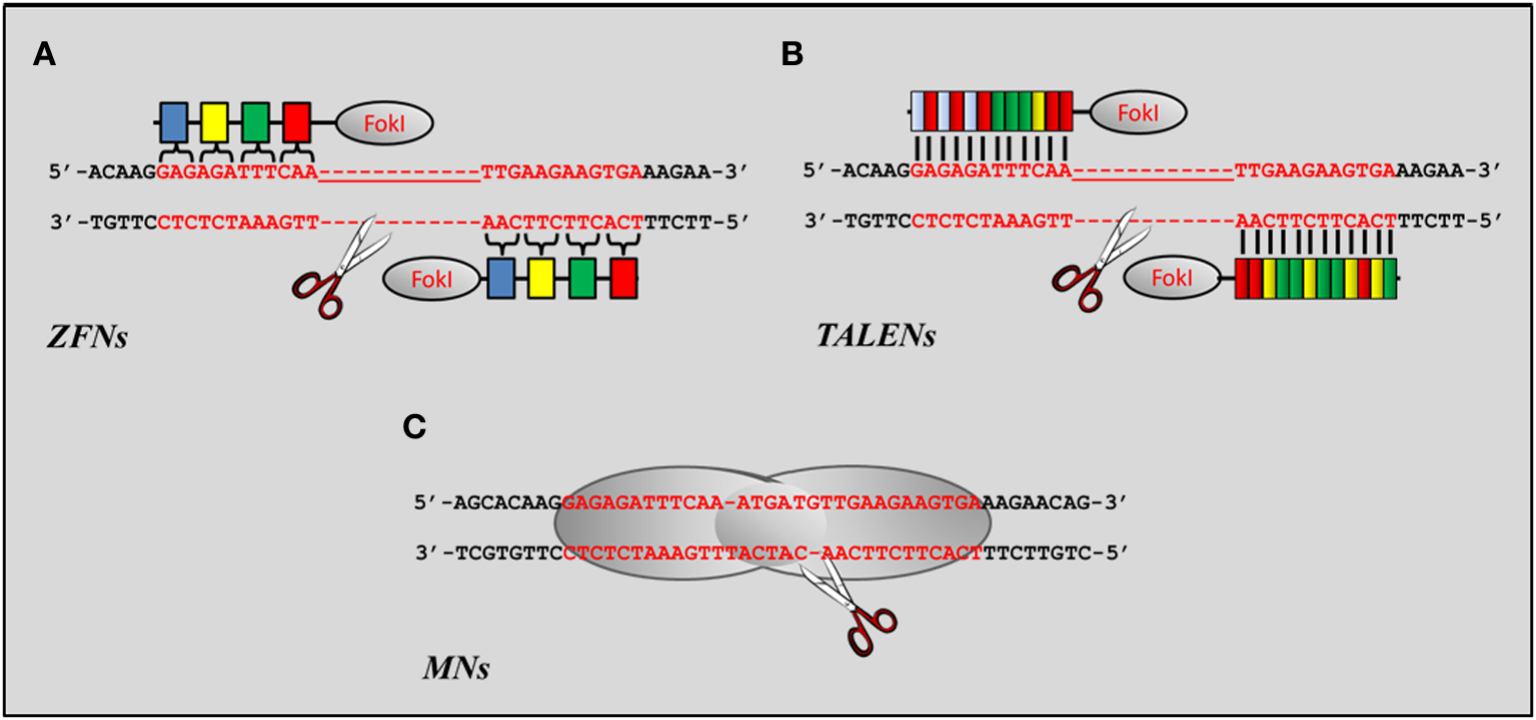
**Nucleases currently used as gene editing tools for muscle disorders. (A)** Schematic representation of a ZFN dimer bound to its target. Each ZFN contains the cleavage domain of *Fok*I linked to a series of zinc fingers each designed to specifically recognize trinucleotide sequences (colored boxes) flanking the cleavage site. **(B)** Schematic representation of a TALEN dimer. Like ZFNs each TALEN contains the catalytic domain of the *Fok*I endonuclease flanked by modules responsible for the recognition of the sequence targeted for repair. Unlike ZFNs, however, each modular repeat binds to a specific bp. Each color represents a module for each of the four nucleotide bases. **(C)** Schematic representation of a LAGLIDADG homing endonuclease (LHE) bound to a DNA target through its catalytic motif. Sequence specificity toward most DNA targets is usually achieved through the association or fusion of protein domains from different enzymes to generate chimeric MNs.

There are three major families of engineered nucleases being employed in gene editing approaches: zinc finger nucleases (ZFNs), transcription activator-like effector nucleases (TALENs), and engineered meganuclease (MNs) (Figure 3). A fourth family termed clustered regularly interspaced short palindromic repeats (CRISPR) has been recently developed as an additional approach to alter genomic sequences at the DNA level (Pauwels et al., 2013). Despite its early stages of development, the use of CRISPR has already proven to be a valid alternative to ZFNs, TALENs, and MNs (Jinek et al., 2012, 2013; Chang et al., 2013; Cong et al., 2013; DiCarlo et al., 2013; Mali et al., 2013; Wang et al., 2013), but its potential for the treatment of neuromuscular disorders has yet to be explored.

**Figure 4:**
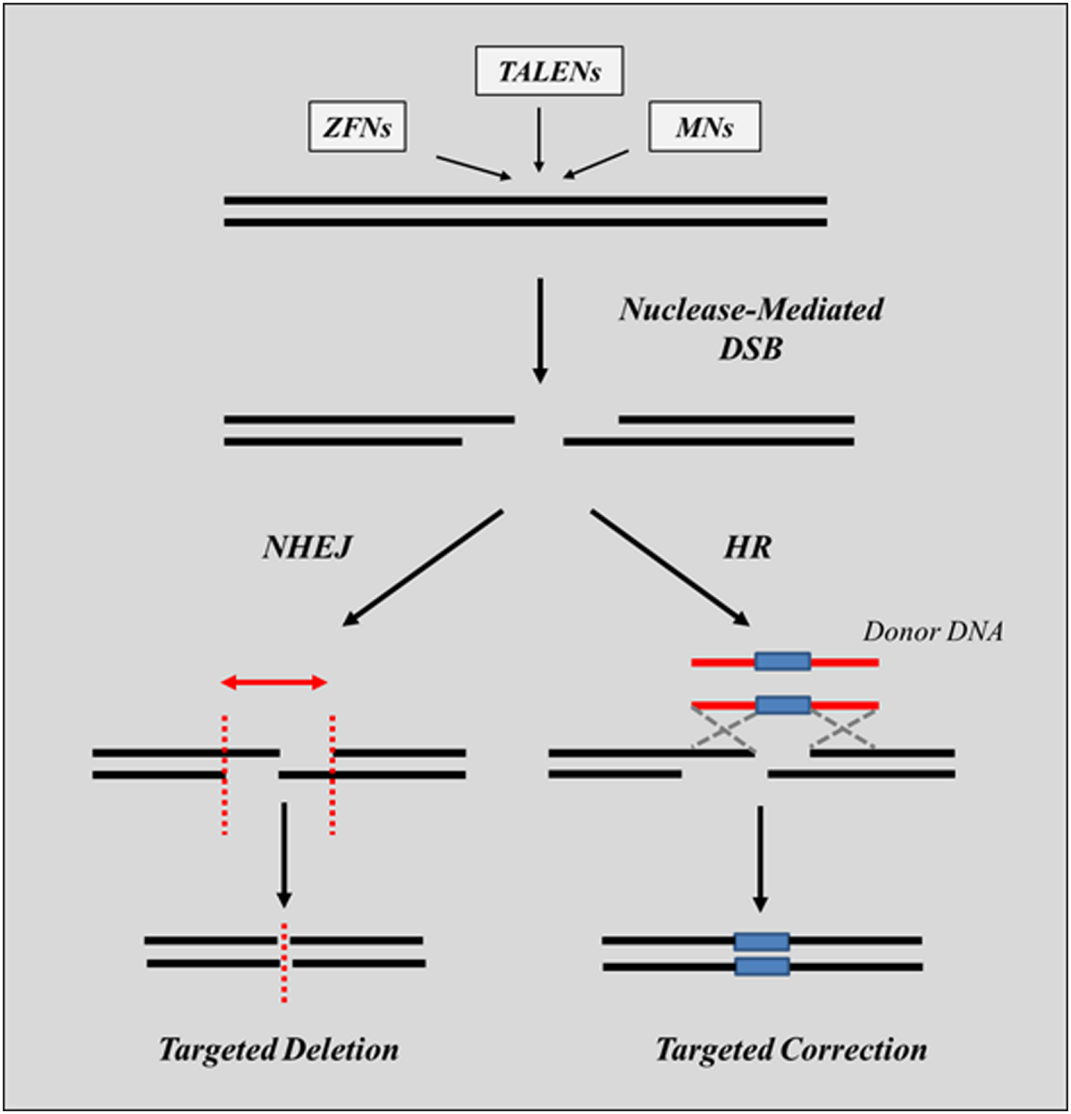
**Gene editing using nucleases**. Upon introduction into the cell and following recognition of the region of the genomic DNA targeted for repair, the binding domains of the nucleases associate and generate a DSB which triggers the activation of specific repair mechanisms. In the absence of donor DNA sequences, the break is repaired by the NHEJ repair pathway by cleaving non-compatible overhang sequences and by joining the ends of the cleaved sequences (targeted deletion). This usually results in deletions of one or more bases (indels). Depending on the type of mutation targeted for repair, these deletions can lead to either, the inactivation of the gene or to the expression of in-frame transcripts encoding for shorter, although still functional proteins. Introduction of single base alterations can only occur in the presence of a donor DNA containing the desired sequence (targeted correction). The process is directed by the HR pathway and requires the exchange of DNA sequences from the donor DNA to the genomic DNA targeted for repair.

The mechanisms of action of nucleases are common to all system and rely on their ability to create a double-strand break (DSB) which is either repaired by NHEJ, or, in the presence of a donor DNA, can be repaired by HR (Figure 4). Several hurdles still need to be overcome before this approach can have a wide-spread use in the context of clinical applications to genetic disorders. Among those, is the limited level of gene editing frequencies achieved in cells, the relative difficulty and time consuming process required to assemble the nucleases *in vitro*, the need to use viral or plasmid vectors to ensure high levels of expression of nucleases in the nucleus required to achieve an effect and the risk of off-target mutations that have been associated with their use (as described in more detail below). Nonetheless, the results reported to date have clearly proven the validity of using engineered nucleases for therapeutic purposes.

### ZFNs, TALENs, and MNs

ZFNs are engineered restriction enzymes obtained by fusing a zinc finger DNA-binding domain to a DNA-cleavage domain originated from the *Fok*I restriction endonuclease (Figure 3A). Each zinc finger domain interacts with 3 bps of DNA and can be engineered to target unique sequences within complex genomes (Bibikova et al., 2003). The DNA-binding domains of individual ZFNs typically contain between three and six individual zinc finger repeats allowing the recognition of sequences of the genome comprised between 9 and 18 bps respectively. Introduction of the DSB is mediated by the dimer formed by the association of two *Fok*I domains. As a result, two ZFNs need to be expressed and bound to opposite strands of the targeted genomic DNA for the cleavage to occur (Figure 3A). Furthermore, each *Fok*I domain needs to be separated by 5–7 bp to allow proper formation of the *Fok*I dimer. To date, ZFNs have been successfully employed to target drosophila (Bibikova et al., 2002, 2003; Beumer et al., 2006, 2008, 2013; Bozas et al., 2009), plants (Shukla et al., 2009; Tovkach et al., 2009; Townsend et al., 2009; Marton et al., 2010; Osakabe et al., 2010; Zhang et al., 2010; Curtin et al., 2011; Qi et al., 2013), *Caenorhabditis elegans* (Morton et al., 2006; Wood et al., 2011), zebrafish (Doyon et al., 2008; Meng et al., 2008; Foley et al., 2009a,b; McCammon and Amacher, 2010; Sander et al., 2011b; Zhu et al., 2011), rat and mouse (Mani et al., 2005; Carbery et al., 2010; Mashimo et al., 2010; Meyer et al., 2010; Cui et al., 2011; Osiak et al., 2011; Chou et al., 2012; Hermann et al., 2012; Bhakta et al., 2013; Shen et al., 2013) and human cells (Alwin et al., 2005; Urnov et al., 2005; Lombardo et al., 2007; Miller et al., 2007; Perez et al., 2008; Hockemeyer et al., 2009; Zou et al., 2009; Holt et al., 2010; Lei et al., 2011; Dreyer and Cathomen, 2012; Handel et al., 2012; Wang et al., 2012).

One of the major drawbacks that has restricted the wide use of ZFNs in the research and clinical settings is the limited availability of DNA-binding domains targeting all trinucleotide combinations and required to guarantee specificity of the ZFN to its target (Desjarlais and Berg, 1992; Pabo et al., 2001), the high cost of purchasing engineered zinc-finger units available in proprietary archives (Pearson, 2008) and, even when DNA-binding domains can be obtained from publically available libraries, the difficulty encountered to assemble and select the ZFNs specific to the desired sequence (Isalan et al., 1997). These limitations have been largely overcome by the introduction of a second generation of engineered nucleases that display similar binding affinity for their target, but higher specificity and relative ease in assembly. TALENs are artificial restriction enzymes generated by fusing a TAL effector DNA binding domain to a DNA cleavage domain (Figure 3B). The DNA-binding domain is generally composed of repeats ranging in number from 33 to 35 amino acids with the exception of the 12th and 13th amino acids which vary for each of the four nucleotides that compose the DNA (Boch et al., 2009; Moscou and Bogdanove, 2009; Mahfouz et al., 2011). Therefore, assembly of the TALEN requires the simple combination of the four possible DNA-binding domains in the order specified by the sequence of the genomic DNA targeted for repair. Similarly to ZFNs, a recognition sequence of 14–20 bp is sufficient to confer specify toward the target site of the genomic DNA, while a separation of 12–19 bp between *Fok*I domains appears to be ideal to guarantee efficient dimerization of the catalytic domain of the nuclease. The relative ease by which these nuclease can be assembled and the much lower cost of producing or purchasing custom vectors expressing a specific nuclease has enable gene editing mediated by TALENs to boom in a relatively short period of time (Cermak et al., 2011; Mahfouz and Li, 2011; Mahfouz et al., 2011; Sander et al., 2011a; Tesson et al., 2011; Wood et al., 2011; Carlson et al., 2012; Li et al., 2012; Liu et al., 2012; Moore et al., 2012; Tong et al., 2012).

A third class of nucleases has also been implemented as potential gene editing tools (Figure 3C). MNs (also known as homing endonucleases) have been developed based on studies that were originally conducted in yeast and that identified mobile elements in the genome (HO and I-SceI) encoded by the mitochondrial genome and responsible for triggering recombination events (Orr-Weaver et al., 1981; Kostriken et al., 1983; Jacquier and Dujon, 1985; Nickoloff et al., 1986; Colleaux et al., 1988). MNs recognize DNA sequences of 12–40 bp in length and are classified in five families based on key sequence and structure motifs. Among those, the LAGLIDADG family is the largest and best characterized one, and the one currently being used for designing MNs (Epinat et al., 2013). The initial studies employed a neomycin resistance reporter gene that was used to demonstrate the feasibility of targeting NIH3T3 and mouse ESCs (Rouet et al., 1994; Smih et al., 1995). Since then, MNs have been successfully applied to induce mutagenesis, recombination or gene targeting in bacteria (Cox et al., 2007; Flannagan et al., 2008), plants (Siebert and Puchta, 2002; Puchta and Fauser, 2013) and mammalian cells (Thermes et al., 2002; Epinat et al., 2003, 2013; Grosse et al., 2011; Izmiryan et al., 2011; Munoz et al., 2011; Menoret et al., 2013). Extensive work conducted on better understanding the mechanisms of action and the amino acid structure of different members of the LAGLIDADG family has enable the construction of chimeric MNs with improved activity and targetability (Seligman et al., 2002; Arnould et al., 2006; Rosen et al., 2006; Smith et al., 2006). Nonetheless, the still limited numbers of sequences that can be targeted combined with the inability of précising direct the recombination of heterodimers necessary to induce target specificity mediated by chimeric MNs (Arnould et al., 2006) has limited its applicability to a large number of genetic disorders.

### Application of engineered nuclease for the treatment of muscle disorders

The use of engineered nucleases to precisely direct genomic alterations in specific genes known to cause muscle disorders has so far been limited to proof-of-concept studies and have focused primarily on determining their applicability for the treatment of DMD (Chapdelaine et al., 2010; Rousseau et al., 2011; Benabdallah et al., 2013; Ousterout et al., 2013). MNs and ZFNs have been used to test the feasibility of activating the NHEJ repair mechanism and of restoring the normal reading frame of a dog microdystrophin gene containing a frame-shift mutation (Chapdelaine et al., 2010; Rousseau et al., 2011). The ability of MNs to induce indels in the dystrophin locus has also been demonstrated through studies aimed at determining the effect of chromatin accessibility on genome editing mediated by MNs (Daboussi et al., 2012). In this study, Daboussi et al designed 37 MNs capable of cleaving different genomic targets. Among those, 5 MNs were shown to efficiently target intronic regions of the dystrophin gene. Although the study was not designed to assess the feasibility of using MNs to restore dystrophin expression, it clearly demonstrated that the dystrophin locus is amenable to gene editing mediated by MNs paving the way for further work aimed at developing effective gene repair strategies to DMD using MNs.

Nuclease-mediated editing of dystrophin gene defects has been recently demonstrated in human cells. TALENs have been tested in primary dermal fibroblasts isolated from a DMD patient harboring a deletion of exons 46–50 (Δ 46–50) and have been used to induce targeted deletions of exon 51 to restore the coding reading frame of the dystrophin gene (Ousterout et al., 2013). Clonal analysis identified a clone with an NHEJ event expected to correct the dystrophin reading frame. When transdifferentiated into myoblasts following ectopic expression of MyoD, cells generated from the clone were shown to form myotubes expressing a dystrophin protein of a molecular weight identical to that predicted as a result of the expression of an in frame transcript lacking exon 46 through 50 of the dystrophin mRNA. The robustness of the approach was further confirmed in immortalized myoblast cells lines isolated from two DMD patients with a deletion of exons 48–50 (Δ 48–50) also treatable by the same pair of TALENs used to correct the Δ 46–50 fibroblasts. In addition to confirm the ability of nucleases of targeting the human DMD gene, these studies have also established the feasibility of using TALEN-mediated NHEJ to target and correct dystrophin gene defects caused by large deletions further extending the applicability of the approach to the majority of the mutations causing DMD.

## Drawbacks and limitations of gene editing mediated by ODNs and endonucleases

As any technology currently being tested and optimized for the treatment of genetic disorders, limitations do exist that may ultimately preclude the use of ODNs or engineered nucleases from entering the clinic for the treatment of DMD. In the case of ODNs, perhaps the major drawback is the low efficiency of the correction process achieved to date. While the use of specific modifications inserted on the oligonucleotides to stabilize the structure of ssODNs (Kayali et al., 2010) or those used to recruit specific repair mechanisms (Bertoni et al., 2009) have proven to significantly increase the gene correction frequencies achieved in muscle *in vitro* and *in vivo*, the frequencies obtained remain low. Other factors are likely to influence the clinical applicability of ODNs including the possibility to induce mutations at regions of the genome different from that targeted for repair, a phenomenon that has not been studied in detail. Additional parameters such as possible toxicity of the ODNs once introduced into patients will also have to be examined.

One of the limiting factors that preclude the use of engineered nucleases, particularly for clinical applications, is represented by the inability to efficiently control the level of expression of the nucleases once delivered to the cell and by the fact that certain endonucleases such as ZFNs and TALENs require the formation of dimers to be active which implies the use of at least two vectors to efficiently express each endonuclease. To date, delivery of endonuclease into the cells has employed, for the most part, the use of plasmid vectors. Although this method of delivery result in efficient expression of the vectors sustained only for a short period of time, potentially the time needed for the nucleases to induce the desired genomic alteration, the level of expression achieved cannot be easily controlled and the efficiency of delivery is limited particularly when targeting stem cells or muscle progenitor cells. An alternative approach is to use lentiviral vectors which have been shown to efficiently transduce stem cells and to be able to achieve high levels of transgene expression. Although effective, these vectors are known to randomly integrate into the genome and therefore they can potentially be mutagenic. This problem has been recently addressed through extensive studies aimed at better characterizing the sequences encoded by the lentiviral vector that direct the recombination process and has led to the development of a new generation of lentiviral vectors unable of integrating (Cornu and Cathomen, 2007; Lombardo et al., 2007). Although safer, these vectors can only achieve limited levels of expression into cells which have been associated with significant lower levels of gene correction frequencies. Promising results have been obtained using adenoviral vectors (Perez et al., 2008; Holkers et al., 2013) and adeno-associated virus vectors which have been shown to drive efficient expression of the nucleases into cells (Porteus et al., 2003; Gellhaus et al., 2010; Metzger et al., 2011). Nonetheless, these vectors still require systems to control their expression once introduced into the cell.

An alternative to the use of viral vectors is the introduction into the cell of mRNA encoding for the nuclease (Geurts et al., 2010; Meyer et al., 2010; Zou et al., 2011) or purified nuclease proteins (Gaj et al., 2012). This method, however, has shown only limited efficacy and is associated with higher costs of production and purification of the endonucleases at the doses required to achieve significant effects.

A major concern that limits the use of endonuclease particularly in the context of therapeutic applications, is the potential off-target effects that have been associated with their use. These effects appear to be related, for the most part, to the lack of binding specificity of the DNA-binding domain toward its recognition sequence (Bibikova et al., 2002; Olsen et al., 2009). As such, cleavage of regions other than those targeted for repair may result in the generation of indels that, once repaired through NHEJ events, could lead to the inactivation of genes. If the inactivation occurs in genes responsible for muscle stem cells maintenance or necessary for the proper function of muscle progenitor cells poses serious safety concerns. Future applications of gene editing targeting and employing stem cells for therapeutic purposes will have to be further refined and issues of toxicity as well as possible side effects will have to be evaluated in detail so as to guarantee safe and long lasting effects.

## Future of gene editing strategies in muscle stem cells

Several parameters need to be considered and optimized before we can reach the stage of designing clinical trials using gene editing approaches that specifically target muscle stem cells. First among all, is the efficacy of the approach being used and the benefits that can be achieved by the different systems being employed. Approaches aimed at correcting stem cells *ex vivo* have clear advantages over systems that target stem cells *in situ* (Figure 1). First, delivery of ODNs or nucleases in cells maintained in culture it is easier to accomplish as it can rely on both chemical as well as physical methods of delivery. For instance, the use of chemical-based reagents such as Lipofectamine™ 2000, Fugene® HD and other of transfection reagents currently in the market, as well as electroporation devices such as Amaxa Nucleofector™ and Neon® Electroporation System have proven to be effective in delivering naked DNA, including ssODNs as well as plasmids encoding nucleases into muscle progenitor cells, SCs, and ESCs (Bertoni and Rando, 2002; Bertoni et al., 2003, 2005; Dekker et al., 2003, 2006; Pierce et al., 2003; Aarts et al., 2006; Flagler et al., 2008; Kayali et al., 2010; Corti et al., 2012; Fontes and Lakshmipathy, 2013). Additionally, viral vectors can be used to express nucleases in cases where chemical and electroporation methods pose a challenge. Another advantage of targeting and correcting stem cells *ex vivo*, is represented by the fact that cells that have undergone repair can be selected, expanded in culture and characterized to ensure that they are safe to use in patients. Importantly, cells to be transplanted into muscle can undergo quality checks to ensure they are devoided of off-target mutations introduced by the ODN or the nuclease in region of the genome other than that targeted for repair. Great progress has been made toward the development of technologies capable of sequencing the entire genome or of studying changes in gene expression at a single cell level. These technologies are likely to become integral part of study design for future clinical trials and will be instrumental to the progress of gene editing technologies toward a safe and effective approach to treat muscle disorders. Finally, clones obtained following selection and expansion can potentially be stored over prolonged periods of times, virtually the lifetime of the patient, and could serve as a reservoir of cells to be used in the event that repeated administrations are required.

The major drawback of *ex vivo* approaches is represented by the difficulty encountered in delivering stem cells into skeletal muscles and the need to target a large number of muscles for the therapeutic approach to be clinically relevant. So far, most of the studies aimed at determining the efficacy of the genetically modified cells to restore muscle function have focused on delivering the cells intramuscularly. Although perfectly suitable for applications aimed at studying the efficacy of the cells being introduced into tissues to engraft and to regenerate muscle, this approach is not applicable in a clinical setting due to the number of injections that would be required to achieve functional effects in patients. Clinical applications to muscle disorders are likely to rely on the use of procedures capable of deliver genetically modified cells systemically. Intravenous or intrarterial injections have been successfully used to deliver Sca-1^+^CD34^+^ (Torrente et al., 2001) and CD133^+^ (Torrente et al., 2004) muscle derived stem cells, mesoangioblasts (Sampaolesi et al., 2006), and pericytes (Dellavalle et al., 2007), but appear to have limited applicability with other cell types. Factors that can promote migration, survival, and engraftment of cells following administration are likely to have important implications for the success of cell-mediated regenerative approaches for the treatment of neuromuscular diseases. Some success has already been achieved following pretreatment of mesoangioblasts with stromal-derived factor-1 (SDF-1) and tumor necrosis factor-α (TNF-α) prior to administration of mesoangioblasts into dystrophic mice (Galvez et al., 2006) and, more recently using inhibitors of junctional adhesion molecule-A (JAM-A) expression, a small immunoglobulin that is located at endothelial and epithelial cell junctions (Giannotta et al., 2014).

Direct delivery of ODNs or nucleases into skeletal muscles may represent a valuable alternative to transplantation of genetically modified cells. In general, this approach will only be able to target endogenous muscle stem cells that are actively participating to the regeneration process such as SCs. Furthermore, it would be applicable only to patients that are at an early stage during the disease process before the reservoir of cells capable of regenerating muscles is exhausted or before their regenerative capacity is compromised as a result of the continue activation typical of muscles that undergo repeated rounds of degeneration and regeneration. Several methods of delivery are currently being optimized and tested for their ability to distribute ODNs and nucleases into different organs and tissues following systemic administration. In the case of ODN-based therapies some success has been achieved using trans-activator of transcription (Tat) protein of the HIV (Frankel and Pabo, 1988; Green and Loewenstein, 1988; Green et al., 1989; Brooks et al., 2005; Bechara and Sagan, 2013). More recently, efforts have been directed toward the identification of short peptide sequences that could be linked to ODNs and used to enhance their uptake through mechanisms of endocytosis and/or direct translocation across the plasma membrane (Joliot et al., 1991; Bechara and Sagan, 2013; Betts and Wood, 2013; Moulton, 2013; Regberg et al., 2013). Some success has been achieved using ODNs tagged to cell penetrating peptides (CPPs) which have been shown to successfully target and distribute ODNs into myofibers following systemic administration (Lescop et al., 2005; Moulton et al., 2007; Ivanova et al., 2008; Wu et al., 2008; Yin et al., 2008; Betts and Wood, 2013). Moreover, the use of CPPs has been implemented in gene editing technologies aimed at enhancing delivery of engineered nucleases with promising results further highlighting the potential of using peptide as carriers to enhance delivery (Nain et al., 2010; Puria et al., 2012; Chen et al., 2013). However, most of the studies thus far have been focused on targeting myofibers and little is known on the ability of CPPs to penetrate SCs or other types of muscle stem cells following systemic delivery.

The main drawback of directly delivering systemically ODNs or nucleases into muscles is represented by the inability to control the repair process once the therapeutic agent reaches it targeted stem cell. As a result, issues of toxicity, off-target effects, and low frequencies of gene repair may limit the beneficial effects achieved. Studies aimed at further refine the efficacy and specificity of the repair process mediated by ODNs or nucleases is likely to have important implications for the success of gene editing approaches.

Independently from the approach used to correct the genetic defect and whether restoration of the missing protein is achieved through delivery of genetically modified cells *ex vivo* or systemic administration of gene editing tools *in situ*, other factors may hamper the efficacy and stability of the repair process. Considerations should be given to possible immune response toward the protein being restored as the result of the therapeutic application. Preconditioning of patients using immunosuppressive reagent as well as administration of chemotherapeutic drugs that are toxic to proliferating cells may be necessary to ensure efficient cell engraftment and rapid clonogenic growth of the transplanted cells.

## Summary and conclusions

The past decade or so has seen an exponential growth in the development of therapeutic applications for muscle disorders specifically designed to target stem cells. Clinical trials are currently undergoing to test the feasibility and efficacy of restoring dystrophin expression in skeletal muscles of DMD patients following systemic administration of mesoangioblasts highlighting the fact that the field is rapidly advancing toward clinical applications for this disease. Approaches aimed at using the patient own stem cells as source for the transplantation procedures has clear advantages over those using heterologous sources of stem cells. As such, it is likely that gene editing approaches will become integral part of future applications to treat muscle disorders using genetically modified cells.

Additional parameters will have to be taken into account and defined before these approaches can enter into the clinic. For instance, gene editing strategies targeting stem cells *ex vivo* will require to refine culturing techniques to ensure that, once explanted, muscle stem cells can be efficiently propagated *in vitro* while maintaining maximal regenerative potential. Furthermore, a better understanding of the mechanisms that regulate stem-cell properties will help redefine and select a specific population of cells that is safe to use in patients without compromising the beneficial effects that can be achieved using the approach. Along the same line, the development of new delivery systems or vectors capable of targeting muscle stem cells *in situ* will be a key to the optimization of gene editing strategies. Ultimately, a key component of preclinical and clinical studies will remain the efficacy and safety of the approach being employed. The trials currently under way for muscle disorders as well as other genetic diseases and the clinical trials that are planned to start within the next few years will be instrumental in determining the key parameters necessary to achieve sustained effects in patients and to ensure the safety and efficacy of the approach being employed. Despite the early stages of gene editing approaches aimed at targeting and correcting stem cells for the treatment of muscle disorders, the results obtained to date are encouraging. Collaborations among different laboratories interested in pursuing these technologies for the treatment of inherited genetic disease affecting muscle could result in advancing gene editing strategies more rapidly and more efficiently into the clinic.

### Conflict of interest statement

The author declares that the research was conducted in the absence of any commercial or financial relationships that could be construed as a potential conflict of interest.
